# Antibacterial effects of children’s and adults’ toothpastes containing different amounts of fluoride: An in vitro study

**DOI:** 10.34172/joddd.40705

**Published:** 2024-03-29

**Authors:** Arthit Klaophimai, Orada Tosrisawatkasem, Sivaporn Horsophonphong

**Affiliations:** ^1^Department of Oral Microbiology, Faculty of Dentistry, Mahidol University, Bangkok, Thailand; ^2^Department of Pediatric Dentistry, Faculty of Dentistry, Mahidol University, Bangkok, Thailand

**Keywords:** Adults, Antibacterial, Children, Fluoride, Lactobacillus casei, Streptococcus mutans, Streptococcus salivarius, Toothpaste

## Abstract

**Background.:**

In recent years, fluoride concentrations in toothpaste for children and adults have increased. However, the effects of different concentrations on bacterial activity have rarely been compared. We aimed to investigate and compare the antibacterial activity of children’s and adults’ toothpaste containing 500, 1000‒1100, and 1450‒1500 ppm fluoride.

**Methods.:**

Three strains of bacteria (*Streptococcus mutans*, *Streptococcus salivarius*, and *Lactobacillus casei*) were cultured in brain heart infusion agar. Thirty commercially available toothpaste products for children and adults containing 500, 1000‒1100, and 1450‒1500 ppm fluoride were selected and tested. Toothpaste’s ability to inhibit bacterial growth was evaluated by agar diffusion assay, in which plates were incubated for 24 hours, and then the diameter of the microbial inhibition zone was measured. Comparisons between children’s and adults’ fluoride toothpastes were made using the Mann-Whitney U test. The association between bacterial growth inhibition and sodium lauryl sulfate (SLS) was analyzed by the chi-square test. A *P* value of <0.05 was considered statistically significant.

**Results.:**

No difference in the inhibition zone was observed for different fluoride concentrations. However, there were significant differences between toothpastes for children and adults, with higher inhibition zones for adults’ toothpastes. Most toothpastes for adults contained SLS, which was associated with antibacterial activity.

**Conclusion.:**

Fluoride concentrations ranging from 500 to 1500 ppm did not affect bacterial growth. The antibacterial activity of toothpastes for adults was significantly higher than that of toothpastes for children, which was mainly attributed to the SLS usually added to adult formulations.

## Introduction

 Dental caries is the most common oral disease affecting children, adolescents, and adults.^1,2^ In a 2017 global survey, the World Health Organization reported 532 million cases of dental caries in primary dentition and about 2.3 billion for caries in permanent dentition.^3^ According to data collected during 1995‒2019, the prevalence of dental caries in Asian children is about 52%‒58%.^1^ This chronic infectious disease is mainly caused by a group of streptococcal species, with multiple contributing factors helping to increase or reduce the risk of caries progression.^4^

 Even though dental caries is prevalent, the disease is preventable. One of the most applicable and common oral health care preventive methods is brushing the teeth with fluoride toothpaste. Brushing teeth causes mechanical removal of dental plaque and food debris, reducing the number of cariogenic bacteria and helping to maintain the balance of normal flora in the oral cavity.^5^ Meanwhile, the ingredients in fluoride toothpaste contribute to caries prevention via two main avenues. The first is via antimicrobial agents added to the toothpaste formula, such as triclosan, sodium lauryl sulfate (SLS), and herbal extracts.^6^ The second is the promotion of remineralization by fluoride.^7,8^ Evidence has shown that tooth brushing with fluoride toothpaste significantly reduces caries increments in both primary and permanent dentition; moreover, the higher the fluoride concentration, the better the remineralization and caries preventive effects.^9^ Additionally, several studies have suggested fluoride itself has antimicrobial properties by inhibiting the activity of bacterial enzymes.^6,10^

 In recent years, the recommended fluoride concentration in toothpaste for caries prevention has shifted from 500‒1000 ppm to 1000‒1500 ppm, which is better to increase preventive efficiency.^11-13^ Consequently, many new commercially available toothpaste products for children and adults have been marketed.^14,15^ However, while many reports have been published on the remineralization effect of 1450‒1500 ppm fluoride,^8,16,17^ few studies have investigated and compared the antibacterial activity of commercially available 1450‒1500 ppm fluoride toothpastes with those having lower fluoride concentrations, and the findings in this regard are still inconclusive.^18,19^ Furthermore, the antibacterial activities of children’s and adults’ toothpastes containing 1450‒1500 ppm fluoride have never been compared. Obtaining this information would further guide dental professionals on the recommendation and selection of toothpastes. Therefore, we aimed to investigate the antibacterial activity of commercially available toothpastes containing 500, 1000‒1100, and 1450‒1500 ppm fluoride and compare the antibacterial activity of children’s and adults’ toothpastes.

## Methods

###  Toothpastes

 The toothpastes were classified into three groups according to fluoride concentration: (*i*) 500 ppm fluoride, (*ii*) 1000‒1100 ppm fluoride, and iii) 1450‒1500 ppm fluoride. The necessary sample size was calculated using G*Power 3.1^20^ with a power of 80% and significance level of 5%, where means and standard deviations were based on previous reports from Randall et al^18^ and Evans et al.^19^ This yielded an estimate of ten samples per group. Accordingly, we selected 30 fluoride toothpastes in each group for this study: 500 ppm, 1000‒1100 ppm, and 1450‒1500 ppm fluoride, which were commercially available (both online and off-the-shelf) in the market in Thailand. For the 500-ppm fluoride group, only toothpastes for children were available, and consequently, only children’s toothpastes were selected. For the 1000‒1100-ppm and 1450‒1500-ppm groups, five children’s products and five adult products were selected. Table 1 presents the details of the toothpastes, including product name, manufacturer, ingredients, and fluoride concentration.

**Table 1:** Details of the toothpastes

**Products and manufacturers**	**Type of toothpaste**	**Ingredients ^®^**	**Fluoride concentration (ppm)**
Angel Blueberry fragrance, Kumho Dental Pharmaceutical (Korean)	Children	Hydrated silica, sodium monofluorophosphate, pyridoxine hydrochloride, licorice extract, calcium glycerophosphate. Sodium PCA solution, green tea extract, concentrated glycerin, rosemary extract, sage extract, D-sorbitol liquid, steviol glycoside, ascorbic acid, grapefruit seed extract, xylitol, purified water, natural.	500 ppm
Aquafresh^®^ Piccoli Denti, GSK consumer healthcare (England)	Children	Aqua, hydrated silica, sorbitol, glycerin, xanthan gum, titanium dioxide, aroma, sodium saccharin, sodium methyl cocoyl taurate, cocamidopropyl betaine, sodium fluoride.	500 ppm
Check-up Banana, Lion Corporation (Japan)	Children	Sorbitol, PG, fragrance (banana), xylitol, sodium polyacrylate, sodium alginate, xanthan gum, carrageenan, sodium citrate, citric acid, palm oil, fatty acid amide, propyl betaine, sodium fluoride, hydroxyethyl cellulose, dimethyl diary 1216, aluminum chloride, cetylpyridinium chloride.	500 ppm
Colgate^®^ Kids, Colgate-Palmolive (Thailand)	Children	Sorbitol, water, hydrated silica, PEG-12, flavor, cellulose gum, sodium lauryl sulfate, tetrasodium pyrophosphate, sodium saccharin, sodium fluoride.	500 ppm
Elmex^®^ Kids, Colgate-Palmolive (Poland)	Children	Aqua, sorbitol, hydrated silica, hydroxyethyl cellulose, cocamidopropyl betaine, olaflur (amine fluoride), aroma, saccharin.	500 ppm
Odol-med3^®^ Erste zahn, GSK Consumer Healthcare (Germany)	Children	Aqua, hydrated silica, sorbitol, glycerin, PEG-6, xanthan gum, titanium dioxide, aroma, sodium saccharin, sodium methyl cocoyl taurate, cocamidopropyl betaine, sodium fluoride.	500 ppm
Giggles Kids, MJ Steps Gmbh (Switzerland)	Children	Aqua, sorbitol, hydrated silica, glycerin, titanium dioxide, aroma, sodium fluoride, sodium saccharin, *Leontopodium alpinum* extract, cocamidopropyl betaine, xanthan gum, sodium hydroxide.	500 ppm
Jordan Milk teeth, Fulijaya Manufacturing (Malaysia)	Children	Aqua, sorbitol, hydrated silica, cellulose gum, flavor, PEG-32, sodium benzoate, sodium saccharin, cocamidopropyl betaine, sodium fluoride, trisodium phosphate, menthol, sodium chloride, cl 42090.	500 ppm
Kindee Organic, Surathin international (Thailand)	Children	Aqua, sorbitol, acrylates/C10-30 alkyl acrylate cross-polymer, xylitol, propanediol, cellulose gum, sodium benzoate, xanthan gum, flavor, PEG-40 hydrogenated castor oil, sodium saccharin, sodium lauroyl sarcosinate, sodium fluoride, potassium sorbate, disodium EDTA, glycerin, calcium phosphoryl oligosaccharides, *Vitis vinifera* (grape) seed extract, aloe bar barbadense (aloe vera) leaf juice, phenoxyethanol, cl 14700, cl 42090.	500 ppm
Oral-B^®^ Stages, Procter & Gamble Manufacturing (Germany)	Children	Water, sorbitol, hydrated silica, sodium lauryl sulfate, trisodium phosphate, cellulose gum, flavor, sodium phosphate, sodium saccharin, carbomer, sodium fluoride, polysorbate 80, cl 42090.	500 ppm
Aquafresh^®^ milk teeth, GSK consumer healthcare (England)	Children	Aqua, hydrated silica, sorbitol, glycerin, xanthan gum, titanium dioxide, aroma, Chondrus crispus (carrageenan), sodium saccharin, sodium methyl cocoyl taurate, cocamidopropyl betaine, sodium fluoride, limonene.	1000 ppm
Godmami^®^ Mild first, cream building (Thailand)	Children	Aqua, glycerin, sorbitol, sodium benzoate cellulose gum, flavor, sodium fluoride, anthemis nobilis flower water, di-panthenol, potassium sorbate, citric acid.	1000 ppm
Jordan New permanent teeth, Fulijaya Manufacturing (Malaysia)	Children	Aqua, Sorbitol, hydrated silica, cellulose gum, flavor, PEG-32, sodium benzoate, sodium saccharin, cocamidopropyl betaine, sodium fluoride, trisodium phosphate, menthol, sodium chloride, cl 42090.	1000 ppm
Pigeon Kids, Neocosmed (Thailand)	Children	Aqua, maltitol, propylene glycol, xylitol, cellulose gum, potassium sorbate, sodium benzoate, sodium fluoride, sodium citrate, glyceryl, caprylate, citric acid, polysorbate 20.	1000 ppm
Orajel Kids^TM^ Mermaid, Church & Dwight (USA)	Children	Water, sorbitol (corn), hydrated silica (mineral), glycerin (vegetable), cellulose gum (tree pulp/cotton seed), cocamidopropyl betaine (coconut-derived), *Stevia rebaudiana* leaf extract (stevia), sodium fluoride, natural flavor.	1100 ppm
DentistePremium care, Siam Cosmeceutical (Thailand)	Adult	Sorbitol, purified water, hydrate silica, sodium lauryl sulfate, xylitol, cellulose gum, mentha piperita oil, sodium fluoride, sodium benzoate, zine lactate, soybean seed extract ferment filtrate, lactoperoxidase, sodium saccharin, ascorbic acid, eucalyptus globulus leaf oil, *Eugenia caryophyllus* flower oil, cetylpyridinium chloride, *Commiphora myrrha* resin extract, *Krameria triandra* root extract, salvia officinalis leaf extract, anthemis nobilis flower extract, *Foeniculum vulgare* seed extract, acacia catechu extract, *Pimpinella anisum* seed extract, *Glycyrrhiza glabra* root extract, *Cinnamomum cassia* bark extract, *Echinacea purpurea* root extract, cl 42080.	1100 ppm
INT-100 Wake me, Pronova laboratories (Thailand)	Adult	Sorbitol, water, silica, sodium laureth sulfate, cocamidopropyl betaine, flavor, menthol, menthyl succinate, cellulose gum, sodium benzoate, sodium saccharin, titanium dioxide, sodium fluoride, potassium sorbate, mannitol, microcrystalline cellulose, mentha piperita oil, sucrose, xylitol, erythritol, ethyl menthane carboxamide, zea mays starch, cyclodextrin, betaine, potassium nitrate, cetylpyridinium chloride, ascorbic acid, tocopheryl acetate, maltodextrin, glycerin, cl 77289, hydroxypropyl methylcellulose, dipotassium glycyrrhizate, *Aloe barbadensis* leaf juice, camellia sinensis leaf extract, cl 42090, sodium citrate, citric acid, cyanocobalamin.	1000 ppm
Listerine^®^ Essential care, Johnson & Johnson consumer (USA)	Adult	Water, sorbitol, hydrated silica, glycerin, PEG-32, sodium lauryl sulfate, cellulose gum, sodium saccharin, eucalyptol, methyl salicylate, thymol, phosphoric acid, menthol, sodium phosphate, xanthan gum, benzoic acid, flavor, *Mentha viridis* (spearmint) leaf oil, disodium phosphate, sodium fluoride, blue 1, yellow 102.	1100 ppm
Mdent Soft cool, Greater poly manufacturing (Thailand)	Adult	Sorbitol, aqua, silica, sodium lauryl sulfate, cellulose gum, mint flavor, disodium phosphate, mineral oil, sodium fluoride, titanium dioxide, menthol, sodium phosphate, sodium saccharin, sodium hydroxide.	1000 ppm
Parodontax^®^ Protect, Neocosmed (Thailand)	Adult	Sodium bicarbonate, aqua, sorbitol, glycerin, hydrated silica, *Mentha piperita* oil, titanium dioxide, sodium lauroyl sarcosinate, silica, aroma, lysolecithin, xanthan gum, sodium saccharin, sodium fluoride, salvia officinalis (sage) oil, cocamidopropyl betaine.	1000 ppm
Aquafresh^®^ Big teeth, GSK consumer healthcare (England)	Children	Aqua, hydrated silica, sorbitol, glycerin, xanthan gum, titanium dioxide, cocamidopropyl betaine, sodium methyl cocoyl taurate, aroma, carrageenan, sodium fluoride, sodium saccharin, limonene, cl 73360, cl 74160.	1450 ppm
Colgate^®^ 3-5 years, Colgate-Palmolive (Poland)	Children	Sorbitol, aqua, hydrated silica, xylitol, PEG-12, cellulose gum, benzyl alcohol, sodium lauryl sulfate, sodium fluoride, aroma.	1450 ppm
Dentiste’ Kids, Siam Cosmeceutical (Thailand)	Children	Sorbitol, purified water, hydrate silica, glycerin, xylitol, xanthan gum, PEG-400, flavor, sodium fluoride, grapefruit seed extract, ascorbic acid, aloe barbadensis leaf juice, copper chlorophyll, *Commiphora myrrha* resin extract, *Krameria triandra* root extract, *Saliva officinalis* leaf extract, *Anthemis nobilis* flower extract, *Pimpinella anisum* seed extract, *Acacia catechu* gum, *Glycyrrhiza glabra* root extract, *Foeniculum vulgare* seed extract, *Cinnamomum cassia* bark extract, *Echinacea purpurea* root extract.	1500 ppm
Odol-med3^®^ Junior zahn, GSK Consumer Healthcare (Germany)	Children	Aqua, hydrated silica, sorbitol, glycerin, PEG-6, xanthan gum, titanium dioxide, aroma, Carrageenan, sodium fluoride, sodium saccharin, sodium methyl cocoyl taurate, cocamidopropyl betaine, Limonene, cl 73360, cl 74160.	1500 ppm
Oral-B^®^ Sugar-free, Procter & Gamble Manufacturing (Germany)	Children	Water, sorbitol, hydrated silica, sodium lauryl sulfate, cellulose gum, flavor, trisodium phosphate, sodium fluoride, sodium saccharin, polysorbate 80, cl 77891, cl 74260.	1500 ppm
Colgate^®^ Total, Colgate-Palmolive (Thailand)	Adult	Glycerin, water, hydrated silica, sodium lauryl sulfate, flavor, arginine, zine oxide, cellulose gum, poloxamer 407, zine citrate, tetrasodium pyrophosphate, xanthan gum, benzyl alcohol, cocamidopropyl betaine, sodium fluoride, sodium saccharin, mica, sucralose, cl74260, cl 77891, cl47005.	1450 ppm
Fluocaril^®^ Original, Greater poly manufacturing (Thailand)	Adult	Water, sorbitol, hydrated silica, glycerin, sodium lauryl sulfate, cellulose gum, cocamidopropyl betaine, flavor, titanium dioxide, sodium monofluorophosphate, sodium benzoate, sodium fluoride, sodium hexametaphosphate, disodium phosphate, sodium saccharin.	1480 ppm
Gum^®^ Ortho, Sunstar Europe (Spain)	Adult	Aqua, sorbitol, hydrated silica, isomalt, PEG-8, lauryl glucoside, aroma, xanthan gum, aloe barbadensis leaf juice, cocamidopropyl betaine, panthenol, sodium saccharin, sodium fluoride, allantoin, sodium chloride, sodium methylparaben, tocopheryl acetate, cetylpyridinium chloride, bisabolol, glycerin, limonene, sodium benzoate, cl 47005, potassium sorbate, cl 420090, *Zingiber officinale* root extract.	490 ppm
Sensodyne^®^ Deep clean, Neocosmed (Thailand)	Adult	Aqua, hydrated silica, sorbitol, glycerin, pentasodium triphosphate, potassium nitrate, PEG-6, sodium lauryl sulfate, aroma, xanthan gum, sodium hydroxide, cocamidopropyl betaine, sodium fluoride, sodium saccharin	1450 ppm
Systema Ultra Care & Protect, Lion Corporation (Thailand)	Adult	Water, sorbitol, hydrated silica, PEG-8, sodium lauryl sulfate, cellulose gum, flavor, cl 77891, sodium saccharin, sodium fluoride, methylparaben, dipotassium glycyrrhizate, o-cymen-5-ol, butylparaben.	1500 ppm

###  Microorganisms and in vitro growth conditions

 The three strains of bacteria used for this investigation were obtained from the American Type Culture Collection (ATCC). The strains consisted of *Streptococcus mutans* (ATCC 25175), *Streptococcus salivarius* (ATCC 13419), and *Lactobacillus casei* (ATCC 334), which were revived and cultured on brain heart infusion agar (BD Difco^TM^ Spark, MD, USA) under anaerobic condition (5% CO_2_) at 37 °C.

###  In vitro investigation of bacterial growth inhibition using agar well diffusion method 

**Figure 1:**
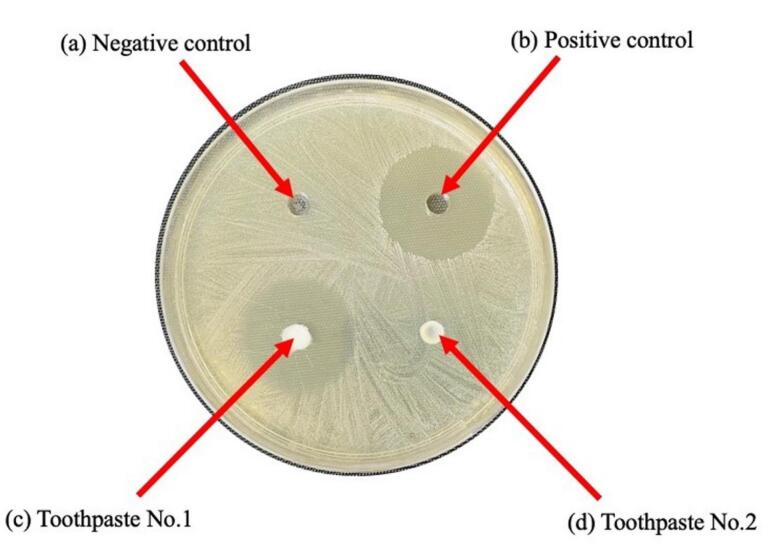
Inhibition zone of toothpastes and controls against *Streptococcus mutans* on an agar plate. a) Negative control (sterile distilled water). b) positive control (0.12% chlorhexidine). c) toothpaste No. 1 (Fluocaril^®^ Original). d) toothpaste No. 2 (Dentiste’ Kids).

 The antimicrobial activity of the toothpastes was investigated using the agar well diffusion assay and measurement of the zone of inhibition. Each bacterial strain was first inoculated in brain heart infusion broth (BD Difco^TM^, USA) and incubated at 37 °C under 5% CO_2_ for 24 hours, after which the inoculums were prepared and adjusted to a turbidity of 0.5 McFarland. Next, 100 µL of each bacterial suspension containing 1.5×10^8^ CFUs/mL was uniformly spread on plated brain heart infusion agar using a sterile cotton swab. A sterile cork borer with a diameter of 6 mm was used to cut four wells into the agar. These wells were seeded with 40 μL of each toothpaste, or 0.12% chlorhexidine and sterile distilled water, respectively, for the positive and negative controls, as shown in Figure 1. Afterward, the agar plates were incubated at 37 °C under 5% CO_2_ for 24 hours, and the diameters of the inhibition zones were measured in millimeters. The test was repeated five times for each tested agent, and the data were presented as mean ± SD.

###  Statistical analysis

 Data were analyzed using SPSS 18 (IBM, Armonk, NY, USA). The Kruskal-Wallis test, followed by pairwise comparisons, was used to compare the three groups of toothpaste with different fluoride concentrations. The Mann-Whitney U test was used to compare children’s and adults’ toothpastes. The chi-squared test was used to examine the association of bacterial growth inhibition with the presence of SLS, an antimicrobial agent, in the toothpaste. A *P* value of <0.05 was considered statistically significant.

## Results

**Figure 2:**
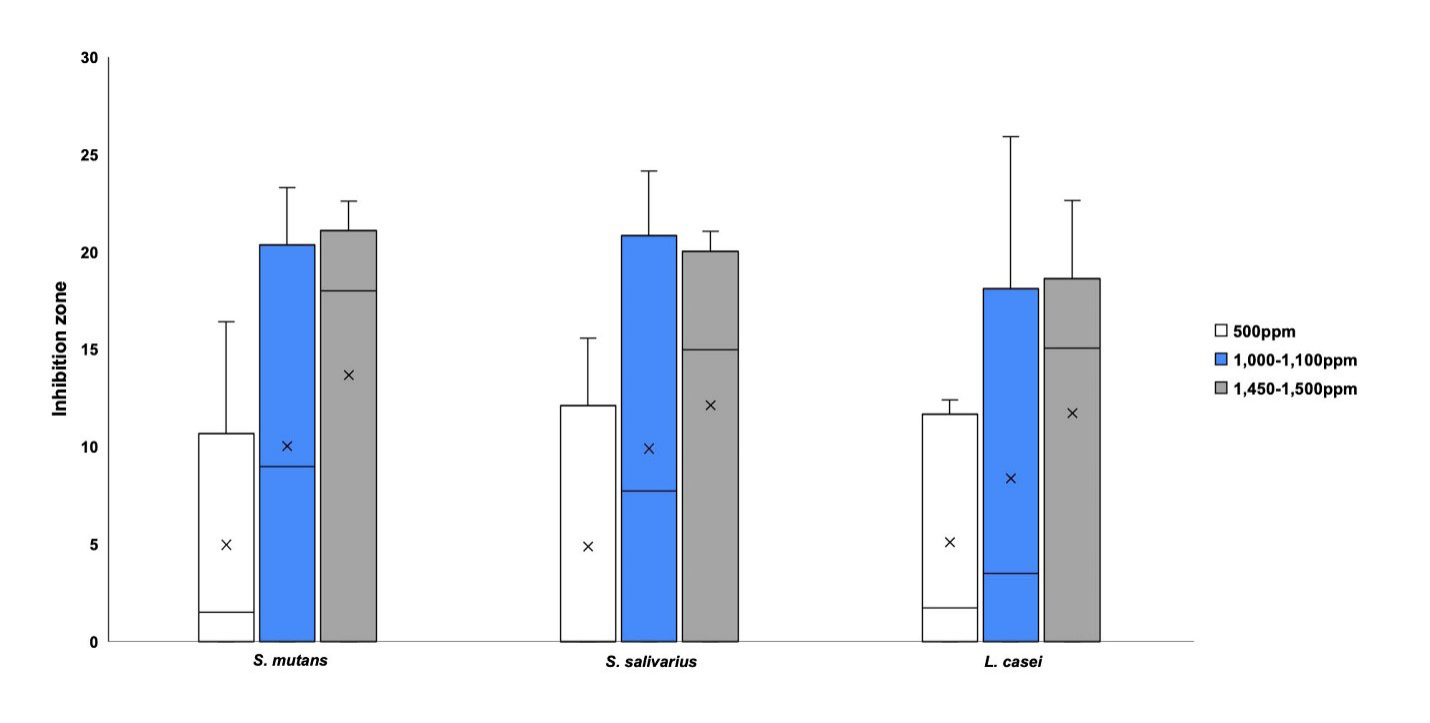
Bacterial growth inhibition of fluoride toothpastes containing 500, 1000‒1100, and 1450‒1500 ppm fluoride. Straight horizontal lines (⎯) represent medians and crosses (×) indicate means. No differences were observed (*P*>0.05).

**Figure 3:**
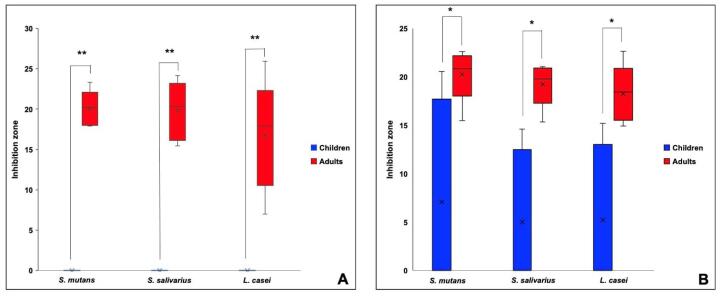
Bacterial growth inhibition of children’s and adult’s fluoride toothpastes. A) Toothpastes containing 1000‒1100 ppm fluoride. B) Toothpastes containing 1450‒1500 ppm fluoride. Straight horizontal lines (⎯) represent medians and crosses (×) indicate means (**P<0.01, *P<0.05).

Table 2 presents the inhibition zone sizes obtained for each of the three strains of bacteria: *S. mutans*, S. *salivarius*, and *L. casei*. Comparisons of the three different fluoride concentrations revealed no significant differences in the inhibition zones for any bacterial strain (*P*>0.05), as shown in Figure 2. However, significant differences were observed when the inhibition zones of children’s and adults’ toothpastes were compared, with children’s toothpastes exhibiting significantly lower inhibition zones for all the three strains (*P*=0.000002, *P*=0.000006, and *P*=0.00003 for *S. mutans*, *S*. *salivarius*, and *L. casei*, respectively). Further comparisons of children’s and adults’ toothpastes having the same fluoride concentration revealed the toothpastes for children consistently had significantly smaller inhibition zones, as illustrated in Figures 3A and 3B. The presence of SLS in the toothpaste ingredients was significantly associated with antibacterial activity (*P*<0.001).

**Table 2:** Zones of inhibition of toothpastes against *S. mutans*, *S. salivarius*, and *L. casei*

**Products and manufacturers**	**Type of toothpaste**	**Fluoride type** **& concentration**	**Zone of inhibition (mm) ± SD**		
			***S. *** ***mutans***	***S. *** ***salivarius***	***L. *** ***casei***
Angel Blueberry fragrance Kumho Dental Pharmaceutical (Korean)	Children	SMFP 500 ppm	0	0	0
Aquafresh^®^ Piccoli denti GSK consumer healthcare (England)	Children	NaF 500 ppm	0	0	0
Check-up Banana Lion Corporation (Japan)	Children	NaF 500 ppm	9.20±1.11	7.95±0.48	11.30 ±1.10
Colgate^®^ Kids Colgate-Palmolive (Thailand)	Children	NaF 500 ppm	16.42±1.07	15.57±0.99	12.38±0.72
Elmex^®^ Kids Colgate-Palmolive (Poland)	Children	Amine fluoride 500 ppm	5.93±0.62	11.62±1.07	11.48±0.99
Odol-med3^®^ Erste zahn GSK Consumer Healthcare (Germany)	Children	NaF 500 ppm	0	0	0
Giggles Kids MJ Steps Gmbh (Switzerland)	Children	NaF 500 ppm	0	0	0
Jordan Milk teeth Fulijaya Manufacturing (Malaysia)	Children	NaF 500 ppm	0	0	0
Kindee Organic Surathin international (Thailand)	Children	NaF 500 ppm	3.04±0.93	0	3.44±0.66
Oral-B^®^ Stages Procter & gamble Manufacturing (Germany)	Children	NaF 500 ppm	15.09±0.41	13.56±0.37	12.20±0.34
Aquafresh^®^ milk teeth GSK consumer healthcare (England)	Children	NaF 1000 ppm	0	0	0
Godmami^®^ Mild first cream building (Thailand)	Children	NaF 1000 ppm	0	0	0
Jordan New Permanent Teeth Fulijaya Manufacturing (Malaysia)	Children	NaF 1000 ppm	0	0	0
Pigeon Kids Neocosmed (Thailand)	Children	NaF 1000 ppm	0	0	0
Orajel Kids^TM^ Mermaid Church & Dwight (USA)	Children	NaF 1100 ppm	0	0	0
Dentiste Premium care Siam Cosmeceutical (Thailand)	Adult	NaF 1100 ppm	23.30±0.43	20.34±1.01	17.92±0.86
INT-100 Wake me Pronova laboratories (Thailand)	Adult	NaF 1000 ppm	18.04±0.82	24.15±0.85	6.99±1.05
Listerine^®^ Essential care Johnson & Johnson consumer (USA)	Adult	NaF 1100 ppm	20.87±0.27	22.25±1.01	18.71±0.68
Mdent Soft cool Greater poly manufacturing (Thailand)	Adult	NaF 1000 ppm	17.92±0.67	16.85±0.96	14.11±0.98
Parodontax^®^ Protect Neocosmed (Thailand)	Adult	NaF 1000 ppm	20.17±0.92	15.43±0.71	25.92±0.91
Aquafresh^®^ Big teeth GSK consumer healthcare (England)	Children	NaF 1450 ppm	0	10.38±0.46	0
Colgate^®^ 3-5 years Colgate-Palmolive (Poland)	Children	NaF 1450 ppm	14.85±0.68	14.62±0.69	10.83±0.37
Dentiste’ Kids Siam Cosmeceutical (Thailand)	Children	NaF 1500 ppm	0	0	0
Odol-med3^®^ Junior zahn GSK Consumer Healthcare (Germany)	Children	NaF 1500 ppm	0	0	0
Oral-B^®^Sugar-free Procter & Gamble Manufacturing (Germany)	Children	NaF 1500 ppm	20.54±1.04	14.69±0.26	15.21±0.77
Colgate^®^ Total Colgate-Palmolive (Thailand)	Adult	NaF 1450 ppm	20.52±1.02	21.06±0.76	18.45±0.75
Fluocaril^®^ Original Greater poly manufacturing (Thailand)	Adult	NaF+SMFP 1480 ppm sodium	21.76±1.06	19.78±1.10	14.90±0.91
Gum^®^ Ortho Sunstar Europe (Spain)	Adult	NaF 1490 ppm	15.49±0.37	15.35±0.99	22.64±1.01
Sensodyne^®^ Deep clean Neocosmed (Thailand)	Adult	NaF 1450 ppm	20.85±0.84	20.74±0.52	19.13±1.02
Systema Ultra Care & Protect Lion Corporation (Thailand)	Adult	NaF 1500 ppm	22.60±0.55	19.21±1.10	16.13±0.63
Positive control	0.12% Chlorhexidine		17.6±0.89	17±0.71	20.6±0.89
Negative control	Sterile distilled water		0	0	0

Note: SMFP, sodium monofluorophosphate; NaF, sodium fluoride

## Discussion

 This study investigated the antibacterial activity of commercial fluoride toothpastes in terms of their ability to inhibit the growth of *S. mutans, S. salivarius, *and* L. casei, *bacterial strains that play roles in biofilm formation and the initiation and progression of dental caries.^21-24^ We found that toothpastes containing 500 ppm fluoride had the smallest inhibition zones, with no significant differences in zone sizes between fluoride concentrations. This finding is consistent with previous reports that fluoride concentration is not correlated with toothpaste antibacterial activity.^18,25^ Some studies have suggested that the antimicrobial function of fluoride requires an acidic environment, which occurs in the oral cavity when the critical pH is reached.^26,27^ Our in vitro investigation maintained a neutral pH for bacterial growth, which might explain our not observing an antibacterial action of fluoride in this study. In contrast, Evans et al^19^ reported commercial toothpastes containing 1450 ppm fluoride to exhibit significantly greater growth inhibition of *S. mutans* and *S. sanguinis* than toothpastes containing 500 ppm fluoride. However, this difference might be attributable to the fact that the 1450 ppm fluoride toothpastes tested in their study also contained antimicrobial agents such as triclosan and sodium bicarbonate, which were not present in the 500 ppm fluoride toothpastes.^6,19^

 We also investigated and compared the antibacterial activity of fluoride toothpastes for children and adults. This study is the first to compare the antibacterial effects of commercially available fluoride toothpastes for children and adults with the same fluoride concentration. Our results showed that the toothpastes for adults resulted in significantly greater inhibition of bacterial growth than those for children, possibly because many commercial toothpaste products for adults contain SLS, a surfactant with antimicrobial properties that interferes with microorganism biological processes and membrane integrity.^28^ Our findings concerning an association between SLS presence and toothpaste’s capacity to inhibit bacterial growth are consistent with previous publications that found toothpastes containing SLS exhibited greater bacterial growth inhibition than those without SLS.^18,29^ SLS creates foam during brushing, leading to the impression of cleanliness^28,30^; however, it also alters taste perception, contributing to a bitter taste after exposure^30,31^ and has been reported to cause some tissue irritation.^28,30^ Distinct from adults’ toothpastes, many toothpastes for children have no SLS due to this taste alteration and chance of irritation. Consequently, fluoride toothpastes for adults demonstrated significantly greater antibacterial activity than those for children.

 Our study had some limitations regarding other factors that could impact bacterial growth. For example, microbial growth and activity are affected by other microorganisms in plaque biofilm and by salivary pH in the oral cavity^26,27,32^; however, this study was an in vitro investigation and inherently limited the influences of such environmental factors. Therefore, absolute data on bacterial growth inhibition in the oral cavity could not be provided.

 Fluoride toothpastes that are branded and marketed for children usually have attractive flavors, smells, colors, and packaging to motivate them to brush their teeth. Children have been reported to prefer toothpastes with a fruity smell and sweet flavor.^33^ However, for those in late childhood and early teens, toothpaste flavor and smell may not significantly affect their brushing decisions and cooperation. Therefore, a recommendation for these groups to use commercially available adults’ fluoride toothpastes may help them gain both remineralization and antimicrobial benefits.

## Conclusion

 Fluoride concentrations ranging from 500 to 1500 ppm did not affect the ability of commercially available toothpastes to prevent bacterial growth. On the other hand, whether a toothpaste is formulated for children or adults was found to influence its effect on bacterial growth, with adults’ toothpastes exhibiting greater antibacterial activity. This inhibitory effect is mainly due to SLS, an antimicrobial agent widely added to adult formulations. Consumers and dental health professionals should be aware of this differential effect and consider it when selecting a toothpaste.

## Competing Interests

 Authors have no conflicts of interest to declare.

## Ethical Approval

 The study was performed according to the Declaration of Helsinki. Neither humans nor animals were used in this study. We used well-known bacterial strains supplied by a business (ATCC) for our research. We do not have the ethical approval code because, according to what we understand, ethical approval was not necessary for this study.

## Funding

 None.
